# Non-invasive magnetic resonance imaging of oils in *Botryococcus braunii* green algae: Chemical shift selective and diffusion-weighted imaging

**DOI:** 10.1371/journal.pone.0203217

**Published:** 2018-08-30

**Authors:** Remco van Schadewijk, Tomas E. van den Berg, Karthick B. Sai Sankar Gupta, Itamar Ronen, Huub J. M. de Groot, A. Alia

**Affiliations:** 1 Leiden Institute of Chemistry, Leiden University, Leiden, The Netherlands; 2 Biophysics of Photosynthesis, Department of Physics and Astronomy, Vrije Universiteit Amsterdam, Amsterdam, The Netherlands; 3 Radiology Department, Leiden University Medical Centre, Leiden University, Leiden, The Netherlands; 4 Institute for Medical Physics and Biophysics, Leipzig University, Leipzig, Germany; Texas A&M University College Station, UNITED STATES

## Abstract

*Botryococcus braunii* is an oleaginous green algae with the distinctive property of accumulating high quantities of hydrocarbons per dry weight in its colonies. Large variation in colony structure exists, yet its implications and influence of oil distribution and diffusion dynamics are not known and could not be answered due to lack of suitable *in vivo* methods. This publication seeks to further the understanding on oil dynamics, by investigating naturally relevant large (700–1500μm) and extra-large (1500–2500μm) sized colonies of *Botryococcus braunii* (race B, strain Showa) *in vivo*, using a comprehensive approach of chemical shift selective imaging, chemical shift imaging and spin echo diffusion measurements at high magnetic field (17.6T). Hydrocarbon distribution in large colonies was found to be localised in two concentric oil layers with different thickness and concentration. Extra-large colonies were highly unstructured and oil was spread throughout colonies, but with large local variations. Interestingly, fluid channels were observed in extra-large colonies. Diffusion-weighted MRI revealed a strong correlation between colony heterogeneity, oil distribution, and diffusion dynamics in different parts of *Botryococcus* colonies. Differences between large and extra-large colonies were characterised by using *T*_*2*_ weighted MRI along with relaxation measurements. Our result, therefore, provides first non-invasive MRI means to obtain spatial information on oil distribution and diffusion dynamics in *Botryococcus braunii* colonies.

## Introduction

Research into biofuel sources is receiving increasing attention as the general public and policymakers become aware of the need to shift from a fossil energy based economy to a more sustainable bio-based economy. The search for new fuels is driven in part by the predicted economic consequences of climate change, but also by the necessity of replacing finite resources [1]. However, first and second generation biofuels have difficulty reaching sufficient economic efficiency, due to the costly conversion steps involved, energy diverted to biomass and a large areal footprint. Therefore, third generation biofuels ideally need to provide direct conversion of CO_2_ into biofuels, avoid conversion losses and also utilize biofuels as an energy sink which would altogether increase yield. Algae, known for their large biodiversity and range of secondary metabolites, could provide a promising solution for this challenge.

Algae-derived biomass has already been suggested as a possible aqua-based alternative to land-based crops [2]. More specific, green algae such as *Botryococcus braunii*, (*B*. *braunii* var. *Showa* [3]) have the advantageous property that they produce oils in lipid bodies, mainly C_30_ to C_34_ botryococcenes, like showacene and isoshowacene [4,5]. Hydrocarbons are present in the form of oils that are similar to those found in petrochemical sources and can be readily refined using hydrocracking [6]. Algaenane complexes comprised of a variety of polymethylated squalenes are also present in *B*. *braunii* race B [7]. In addition, *B*. *braunii* is considered to be an important contributor to petroleum generation, being linked to Torbanite and Coorongite oil shales [8–10].

Oil in *Botryococcus* is believed to serve multiple purposes, including buoyancy control that allows for floatation [8,11]. Furthermore fatty acids excreted by some strains have allelopathic effects on other phyto algae and cause fish death during blooms [12]. Much attention has been focused on improving the relatively slow growth of *Botryococcu*s strains [13,14]. Hydrocarbon accumulation is intimately linked to cell division, being important for cell-cell cohesion and structure, thus influencing growth patterns [15]. Recent work on B. *braunii* Race A indicates that lipid bodies are formed in the cytoplasm, with hydrocarbon synthesis reaching its maximum during septum formation [16]. Suzuki *et al*. proposed the central role of outer cell wall layers in the formation of an intercellular matrix, formed by successive cell divisions [17]. These findings, combined with accumulation of oil in the extracellular spaces, have captivated research attention focused on using *B*. *braunii* for biofuels for decades, with publications starting to accumulate from the 1980’s onwards. The interest increases explosively from the 2000’s up to now.

Until now detailed information on colony anatomy is restricted to small sized colonies (30–200 μm) [16,18,19]. However, *B*. *braunii* shows a large diversity in colony size under natural conditions, with values of 30–2000 μm being reported in the wild [20]. There also exist naturally occurring algal blooms with colony sizes of up to 1500 μm [21]. Bloom formation is especially notable in water reservoirs where blooms were reported up to 1500 metric tonnes in terms of biomass [21,22]. A detailed and comprehensive picture of *B*. *braunii* physiology and its oil accumulation characteristics under natural conditions is still missing. It is unknown how the large variation in the colony size is linked to oil accumulation in localized domains and whether diffusion characteristics are influenced by colony structure. Observing the anatomical structure of various colonies, together with direct *in vivo* mapping of oil domains, would help us to understand the link between colony structure and oil accumulation behaviour. These observations could provide insight into the functions and mechanisms underlying these large variations in colony structure. Furthermore, distribution of different types of oil within larger sized colonies could be useful for the prediction and optimisation of production yields. The unique properties of *B*. *braunii* make experimental studies challenging, especially considering the copious mucilage exuding from the colonies and the large range in colony size [21]. Optical microscopy, including staining, FLIM, etc., allow for high-resolution study of colony anatomy but relies on invasive cross sections, and the metabolite composition in localized domains within intact colonies cannot be approached. Solution state NMR and HR-MAS NMR have been utilized to determine lipid contents extracted from *B*. *braunii* colonies [23,24]. However, localized information about lipid and metabolite distribution and their relation to colony structure cannot be obtained in intact live colonies utilising these techniques. Among other strategies to overcome these limitations, confocal Raman spectroscopy has seen application in *B*. *braunii* race B to image specific hydrocarbons, although with low resolution [25].

This study, therefore, aims to optimize and exploit non-invasive Magnetic Resonance Imaging (MRI) in conjunction with chemical shift imaging and spectroscopy to study variation in colony structures and *in vivo* mapping of oil domains in live naturally relevant sized colonies, such as large (700–1500 μm) and extra-large (1500–2500μm) colonies. Furthermore, diffusion MRI has been exploited to get insight into the diffusion dynamics. Our results show that MRI at high magnetic field (17.6T) allows for mapping oil/hydrocarbon distribution in *B*. *braunii* directly in live colonies *in vivo*, thereby avoiding many of the elaborate and invasive analytical steps typically required to investigate algae. This study also provides for the first time non-invasive MRI means to obtain spatial information of oil distribution, and diffusion dynamics *in vivo*.

## Results

**Fig 1:**
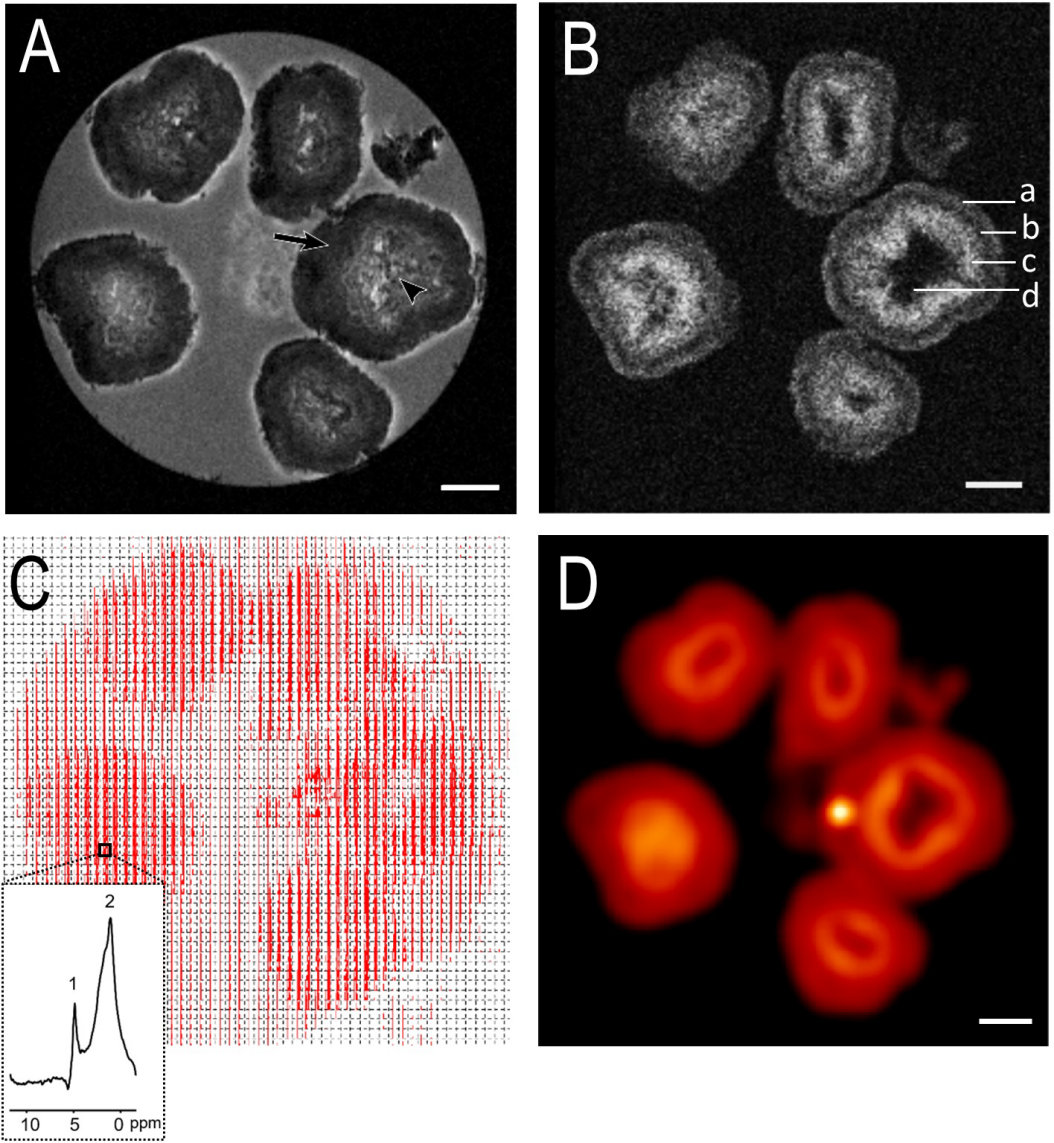
High resolution μMRI images of *B*. *braunii* colonies measured at a magnetic field of 17.6T. (A) Axial image of colonies ranging in size from 700–1500μm. Images were obtained using the multislice multiecho (MSME) pulse sequence by averaging 4 echo images (average echo time, 13 ms; repetition time, 1500 ms; field of view (FOV), 5.0x5.0 mm and number of averages, 32) with a resolution of 19.5x19.5x100 μm^3^. Black arrowheads show central core and black arrows represent a clear dark band of 200–300 μm thickness surrounding the colonies. (B) Chemical Shift Selective Imaging (CSSI) of oil/hydrocarbon resonances. Imaging parameters were: repetition time, 1500 ms; echo time, 8.3 ms; number of averages, 64; resolution, 19.5x19.5x250^3^ μm. Receiver bandwidth used was 100 kHz. Excitation pulse of 600 Hz wide at -3.35pm in respect to water resonance was used. High oil containing region (region a, c) and low/no oil region (region b, d) can be clearly seen. (C) Matrix display of chemical shift imaging spectra. CSI data was recorded with a repetition time of 1100 ms, echo time of 12 ms and slice thickness was 0.25 mm. Total averages were 62. Resolution obtained was 156x156x250 μm^3^. Spectral width used was 10 kHz (13.33 ppm) and 32x32 matrix was reconstructed into 64x64 voxels. Inset: Representative spectra of single voxel showing residual water (1) and fat resonances (2). The main–(CH_2_)_n_−signal in colonies is centred around 1.3 ppm, with side lobes from–(CH_2_)_n_–CH_3_ up-field and -CH_2_-CH = CH-,–CH_2_–CH_2_–COOR extending downfield. (D) CSI voxel intensity thresholding. Signals between 0.80 to 1.25 ppm corresponding to fat were chosen to reconstruct CSI images and overlaid with corresponding *T*_*2*_-weighted MSME image using the Bruker CSI Visualisation Tool. Scale bar: 500 μm.

To cover the wide range of colony sizes exhibited by *B*. *braunii*, a dedicated Micro5 probe with a built-in strong gradient (2 T m^-1^) was used. A 5mm volume RF coil with large vertical linear B1 range, which was specifically designed for a 17.6T magnet, was selected to obtain sufficient resolution and signal to noise ratio. Fig 1A shows a high-resolution morphological image of the colonies obtained by the Multi Slice Multi Echo (MSME) sequence. A detailed view of successive axial slices through the colonies is shown in supplementary figure (S1 Fig). The image slice in Fig 1A shows multiple colonies with varying size but with highly similar colony structure. The colonies are ranging 700–1500 μm in diameter, and have moderately heterogeneous centres (black arrowhead). Henceforth we define these colonies as ‘large colonies’ to distinguish them from small conies typically studied in literature (size 20–200 μm). The edges of these large colonies show an irregular surface area, with small extrusions representing botryoidal ‘bunch-of-grapes’ growth patterns. This is in line with morphological data observed for colonies of *B*. *braunii* with a combination of optical and electron microscopy [15]. There is a dark band of 200–300 μm thickness near the surface of the colonies (black arrow). This band is most likely comprised of cells in the extracellular matrix, in which living cells occur predominantly near the surface. In this respect, recently Wijihastuti et al. [26] have shown that when *B*. *braunii* is grown as biofilm, the living cells are confined to a surface layer of 20–60 μm.

### Chemical Shift Selective Imaging

The Chemical Shift Selective Imaging (CSSI) Sequence was utilised to resolve the spatial distribution of oil and water in *B*. *Braunii* large colonies (Figs 1B and S2).

Though oils are generally present in very high proportion, other compounds such as algaenane share similar resonant frequencies as that of botryococcenes oils, causing them to be registered jointly in the CSSI images. In this work we use the term oil to describe this hydrocarbon fraction, with it being understood that it is a part of a complex mixture of hydrocarbons and their derivatives. In the image slice shown in Fig 1B, oil is localized in two defined concentric rings, that are the cross sections of an outer layer 100–150 μm thick (region a) and an inner layer 200–300 μm thick (region c). The outer layer can be attributed to a hydrocarbon rich region of the extracellular matrix surrounding living cells [12]. The thicker inner layer shows a high signal intensity of oil that can be attributed to the hydrocarbon cross-linked network associated with cell remnants comprised of layers of mother cups [21]. In addition, the thicknesses of both the hydrocarbon containing layers are uniform over the sample, across different colonies and over the range of colonies size (700–1500 μm) (Fig 1B). The region of 100 μm thick (region b) between the two oil layers corresponds to a transition region between the living cells (layer a) and the cell remnants (layer c). Interestingly, in contrast to the outer layers, oil was not detected in the centres of the colonies. It is unlikely that oil is present in this region and CSSI sequence could not detect it due to line broadening that may arise due to presence of paramagnetic ions, since the MSME imaging works well in these areas (Fig 1A).

The contrast between oil rich and oil poor regions is greater for large colonies compared to smaller colonies (region d) (Fig 1B). Localisation of the water signal by CSSI reveals the presence of high concentrations of water in the centre, but not in the outer layers of the colony (S2 Fig). In addition, in many colonies the distribution of water in the centre region is inhomogeneous and gradually varies, *i*.*e*. without distinct boundaries (S2 Fig).

### Chemical shift imaging

The content and distribution of oil were further analysed spectroscopically by chemical shift imaging (CSI). Chemical shift imaging exploits differences in the local magnetic field experienced by protons to capture localized spectra in a 2D or 3D matrix. Because the resonance frequency of protons depends on the local magnetic field seen by the proton, protons in lipid molecules resonate at different frequency compared to those in water molecules. CSI utilises this principle of chemical shift for localised spectroscopic imaging, by forgoing the readout gradient used in imaging (including CSSI) and instead incorporating an additional phase encoding step. The pulse sequence then captures a full spectrum for each encoded voxel. CSI therefore allows mapping the spatial distribution of hydrogen nuclei associated with water or with lipid molecules. CSI has an advantage over single- or multiple-voxel localization techniques since there is no chemical shift artefact problem in CSI [27]. Because of this, it is useful for high-field *in vivo* MRS applications in which the chemical shift dispersion increases linearly as a function of B_0._ CSI data are presented as a matrix array of spectra in Fig 1C. In a representative spectrum, taken from the highlighted area in Fig 1C, two major peaks are visible belonging to water (1) and oil (2) within colonies (inset Fig 1C). The main–(CH_2_)_n_−signal of lipid in colonies is centred around 1.3 ppm, with side lobes from–(CH_2_)_n_–CH_3_ up-field and -CH_2_-CH = CH-,–CH_2_–CH_2_–COOR extending downfield. A map of oil or water can be displayed in colour and overlaid on an MR image of the same slice. The CSI image of oil signal integration is depicted in Fig 1D, revealing that indeed all large colonies (700–1500 μm) exhibited a double oil layer (a, c) and very low oil at the centre of the colony (d). The inner oil ring appears to be higher in oil concentration as compared to outer oil ring as seen in both CSSI and CSI measurements (Fig 1B, 1C and 1D). Thus, CSI is not only able to accurately measure protons of lipid/oil but also assessed its distribution and relative intensity in localized domains in *B*. *braunii* colonies.

### Extra-large colonies show a significantly heterogeneous structure

**Fig 2:**
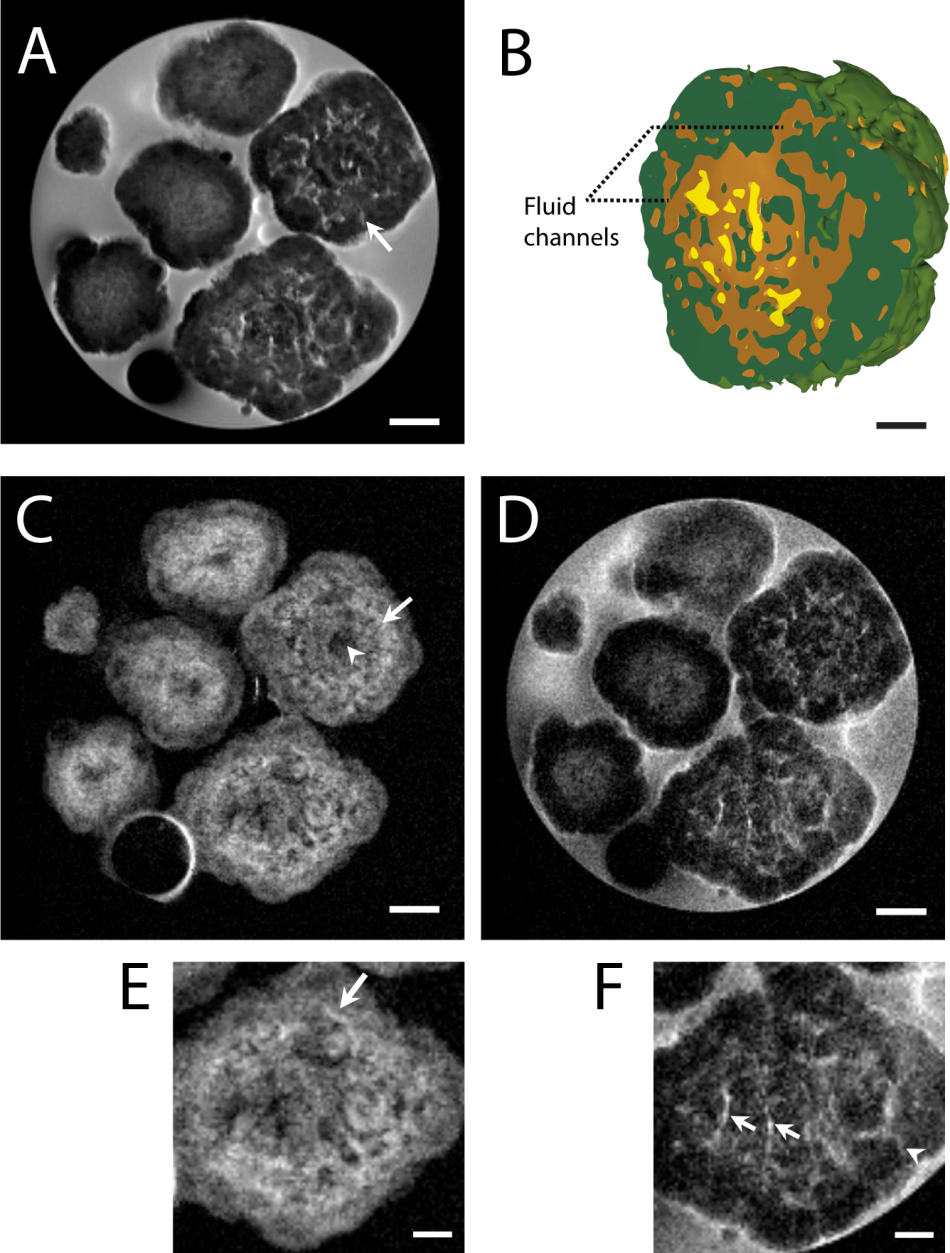
Diversity in colony types visualised at high magnetic field (17.6T). (A) Image was obtained using 2D MSME pulse sequence (echo time, 13 ms; repetition time, 1500 ms; number of averages, 128) with an in-plane resolution of 19.5 x 19.5 μm. Large and extra-large size colony types can be discerned. Arrow indicates a fluid channel. (B) 3D-volume reconstruction of extra-large colony based on voxel intensity thresholding, showing presence of fluid channels. (C) Spin echo CSSI of oil/lipids showing a pattern of low (arrowhead) and high (arrow) oil containing regions. Oil appears absent in the centre of the colony. (D) Spin echo CSSI of water signal. Water channels are visible in extra-large colonies. (E) Enlarged area of (C) showing possible septum (white arrow). (F) Enlarged area of (D) showing fluid channels (white arrow), some of them reaching the colony surface (white arrowhead). Scale bar: 500 μm (A,C,D) and 250 μm (B,E,F).

Within samples of *B*. *braunii* cultures, some extra-large colonies are also visible (ranging between 1500–2500 μm diameter) (Fig 2A). These colonies show a significantly heterogeneous structure especially at their centres, as compared to ‘large sized’ colonies (<1500 μm). Henceforth we refer these colonies as ‘extra-large colonies’. Interestingly, fluid channels reaching the surface are observed in these extra-large colonies, and are indicated with a white arrow in Fig 2A. The 3D intensity reconstruction made from one of the extra-large colonies shown in Fig 2B reveals heterogeneity in the colony and the presence of channels reaching the surface.

Analysis of the oil distribution in extra-large colonies revealed that oil was spread throughout colonies, but with large local variations (Fig 2C). The double ring structure as observed for smaller colonies, was also present in extra-large colonies, but the hypo-intense region between the rings was less clear. In contrast to smaller colonies, the central part of the extra-large colonies contains a pattern of low (arrowhead) and high (arrow) concentrations of oil. Conversely, water distribution for the extra-large colonies shown in Fig 2D indicates that water is distributed throughout the colony in the form of channels, with some of these channels or interfaces reaching the colony surface (Fig 2F). Curiously, some hyper intensities were observed in the oil CSSI image, which can be speculated to be a part of the interface or septum between sub-colonies (Fig 2E, arrow).

A false colour overlay image generated from Fig 2C and 2D revealed distribution of the water and oil signal (S3 Fig.). In general, local highs in oil are correlated with a local depletion of water and vice versa. Additionally, the area in between the oil rings of a large colony appears dark in S3 Fig, implying that both oil and water signals are weak in this area (white arrow).

### *T*_*1*_ and *T*_*2*_ Relaxation properties of *B*. *braunii* colonies

**Fig 3:**
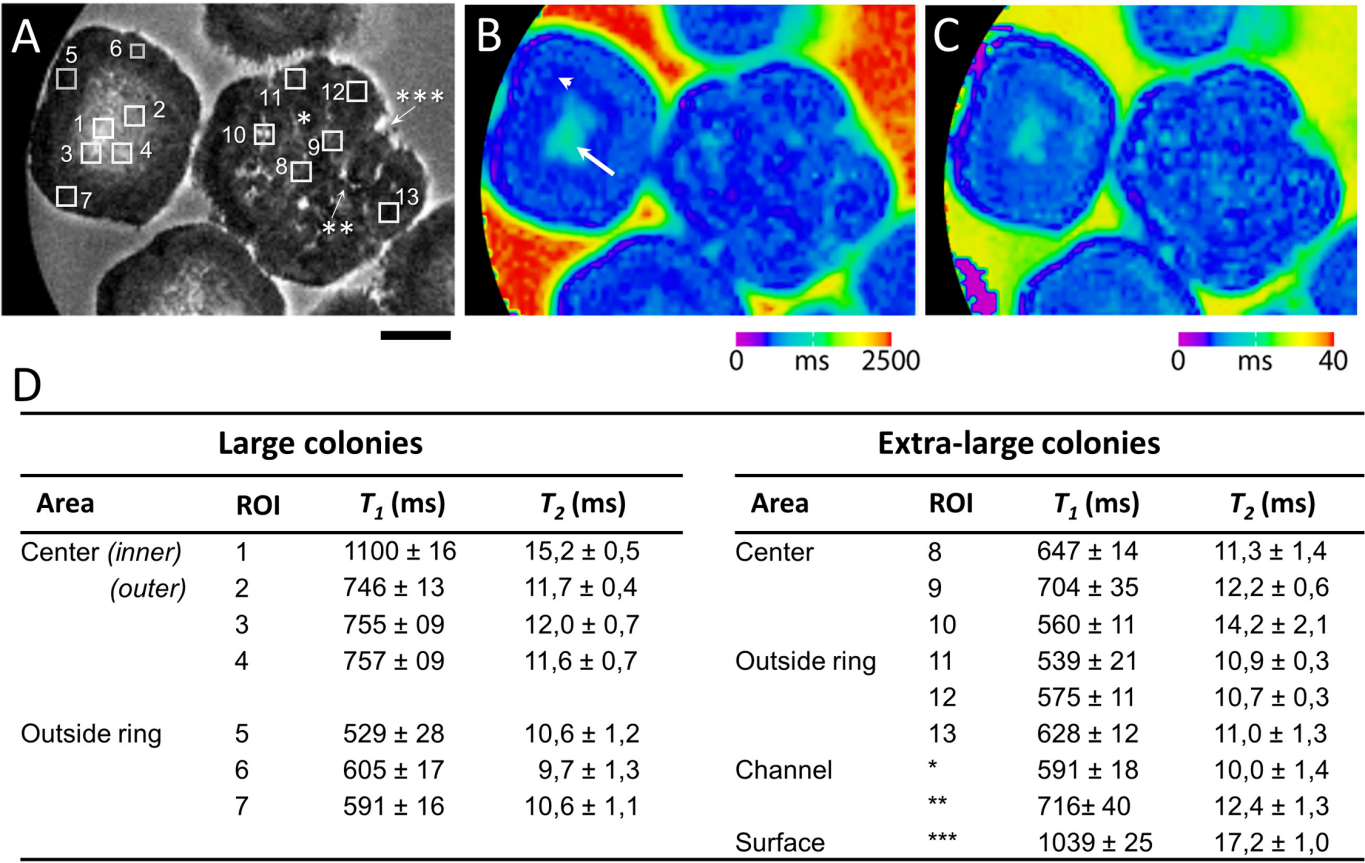
*T_1_* and *T_2_* relaxometry and mapping of *B*. *braunii* colonies. Relaxation measurement was performed using RAREVTR sequence (TR-array, 5500–200 ms; TE, 27–4.5 ms; number of averages, 16; matrix size, 128 x 128; FOV, 0.5 x 0.5mm; resolution was 39 x 39 x 250 μm^3^). (A) A representative image showing regions of interest (ROI) placed on two representative colonies (one large and one extra-large size colony) for calculating *T*_*1*_ and *T*_*2*_ relaxation times. Scale bar: 500 μm. (B) *T*_*1*_ Map derived from RAREVTR sequence, showing the region of high (white arrow) and low *T*_*1*_ (white arrowhead). Colour scale was generated with Paravision ‘colour 256’ scheme which ranges from 0 to 2500ms. (C) *T*_*2*_ Map Derived from RAREVTR sequence showing a sharp edge of low *T*_*2*_ surrounding all colonies (black arrow). Colour scale ranges from 0 to 40ms. (D) Tabulated data of *T*_*1*_ and *T*_*2*_ relaxation times calculated from ROI indicated in (A).

Morphological variation and differences in oil concentrations between colonies can also be reflected in proton longitudinal (*T*_*1*_) and transverse (*T*_*2*_) relaxation properties, which can be used as surrogate biomarkers for a colony type. In order to evaluate relaxation variations within colonies, several representative regions of interest (ROI) were selected within a large sized (top left) and in an extra-large colony (middle) (Fig 3A). The *T*_*1*_ and *T*_*2*_ relaxation times were calculated and compared with observed structural details shown in tabular form in Fig 3D.

The longest *T*_*1*_ was observed at the inner centre of the large sized colony, (region 1, ~1100 ms), while the outer central region shows significantly shorter *T*_*1*_ (regions 2–4, ~753 ms). Hence, it can be concluded that the composition of the central water-containing region of these colonies is heterogeneous. The outer layers of these colonies (regions 5–7) exhibit shorter *T*_*1*_ (average ~578 ms). The difference in *T*_*1*_ for the central and surrounding regions is also clearly visible in *T*_*1*_ map depicted in Fig 3B, showing a long *T*_*1*_ for a confined light blue area in the centre of the large sized colony (white arrow) and significantly shorter relaxation times for the surrounding regions (dark blue, white arrowhead).

Extra-large colonies exhibited a variation in the distribution of *T*_*1*_ relaxation time. The *T*_*1*_ varied between ~560 ms to ~704 ms (regions 8–10) within the central part of the colony. The heterogeneity of *T*_*1*_ in the central part is more pronounced in extra-large colonies as compared to large colonies. The colony outside ring *T*_*1*_ was very similar in comparison to large colonies, ranging from ~539 to ~628 ms (regions 11–13).

Fluid channels within the extra-large colony have varied *T*_*1*_ relaxation times as denoted by asterisks in Fig 3A (~591 ms and ~716 ms). Based on these values, it can be concluded that there is possibly variation in fluid composition corroborating the findings from CSSI imaging. However, visibility of fluid channels depends on both *T*_*1*_ and *T*_*2*_ contrast, since the image intensity is not directly correlated with the *T*_*1*_ relaxation time.

The transverse relaxation time or spin-spin relaxation time, *T*_*2*_, is a specific attribute of spins, which depends on their surroundings. Interaction between spins, for example, coupling to neighbouring nuclei, destroys the phase coherence; therefore, the *T*_*2*_ relaxation time can be a sensitive indicator of the variation in the microenvironment within a colony volume. The centre of the large sized colony exhibited the longer *T*_*2*_ (15.2 ± 0.5 ms) as seen by the blue-green region in Fig 3C. In contrast, the outer central regions (region 2–4) have significantly lower *T*_*2*_ (~11.6–12.0 ms). The edge of the colony show lower *T*_*2*_ relaxation time (ranging from ~9.7ms to ~10.6 ms (region 5–7), which is also reflected by a dark purple ring seen in the *T*_*2*_ map (Fig 3C, black arrow).

In extra-large colonies, the *T*_*2*_ times measured in the centre show high variation (ranged from ~11.3 ms to ~14.2 ms). In contrast, in large size colonies, *T*_*2*_ relaxation time spread was smaller in the centre (region 2–4), confirming that the sample composition may be more homogeneous in these colonies. On the other hand, the *T*_*2*_ relaxation times in the centre of the extra-large colonies was significantly different, especially due to the presence of fluid channels (* and **). Overall, these results confirm the imaging data that extra-large colonies are more heterogeneous in appearance as compared to normally sized colonies. In general the variation in *T*_*2*_ over different region is rather small, which could be due to the fact that *T*_*2*_ values of different fraction (a mixture of water and hydrocarbon) within a voxel are averaged. In addition, high level of hydrocarbons do not appear to translate to longer *T*_*2*_ relaxation times. This may possibly be a result of enhanced rate of relaxation of hydrocarbons due to interactions with the local environment.

A hyper-intense ring is visible at the surface of all the colonies as seen clearly in Fig 3A. This ring was especially visible under TR ≤ 1500 ms. This hyper-intense ring is also reflected in the *T*_*1*_ map (Fig 3B) and to a lesser extent present in the *T*_*2*_ map (Fig 3C). In the *T*_*1*_ map, the ring appears as a transition zone with increasingly higher *T*_*1*_ further away from the colony. The transition zone (***) contained a long *T*_*1*_ of ~1039 ms. It is likely caused by diffusion mediated interactions between the surrounding medium and the exopolysaccharide fibrillary sheath of *B*. *braunii* [28,29].

### Diffusion behaviour is correlated to colony size

**Fig 4:**
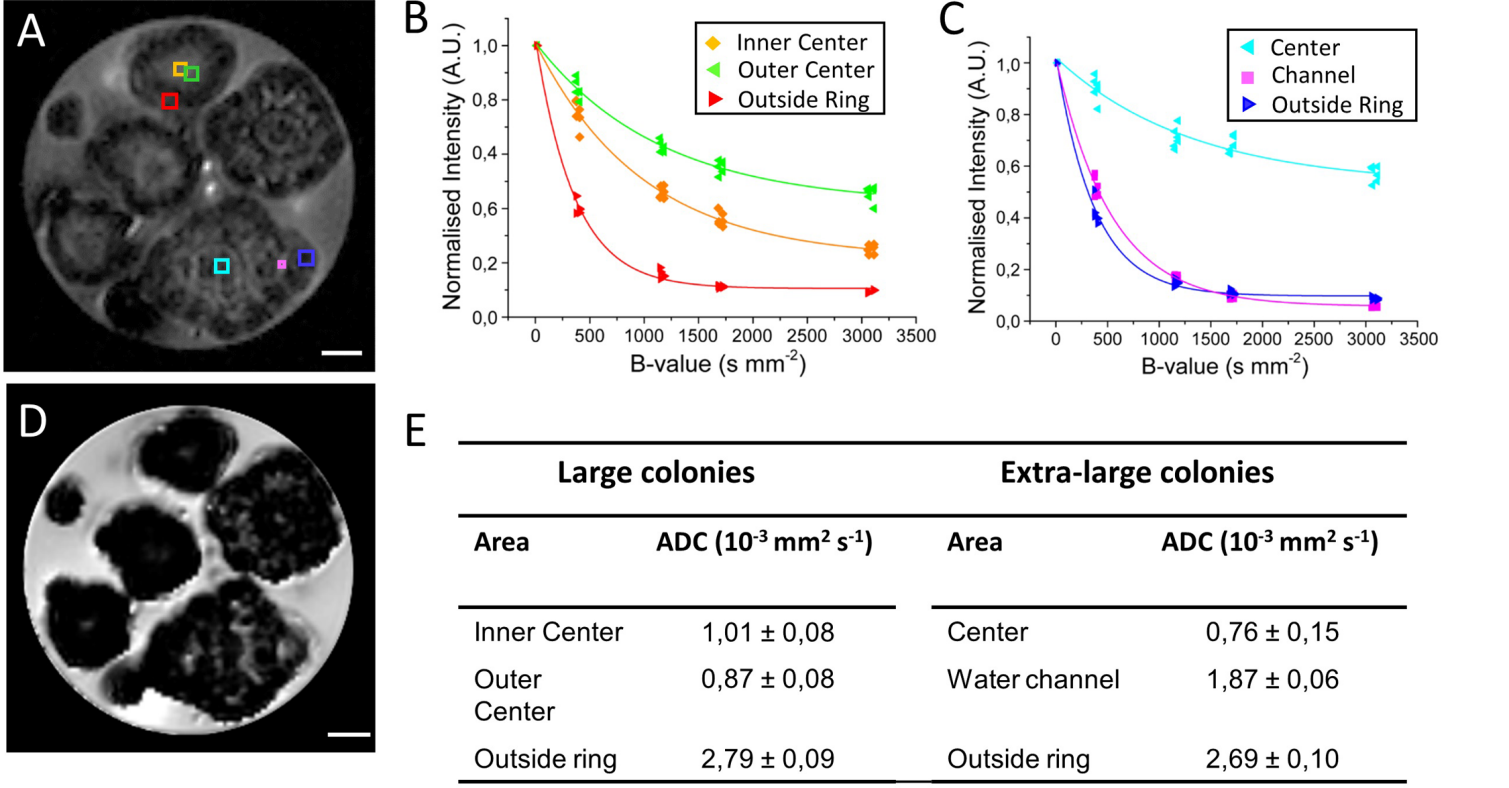
Diffusion is strongly correlated to colony size. Diffusion measurements were performed using a spin-echo pulse sequence containing a pair of mono-polar diffusion-sensitising gradients (TR, 1500 ms; TE, 10.1ms; 16 averages; diffusion gradient duration 2.5 ms and gradient separation of 5 ms; effective B-values range: 14, 406, 1182, 1723, 3123 s/mm^2^). (A) A diffusion image showing regions of interest (ROI) placed on two representative colonies (one large and one extra-large sized colony) for calculating apparent diffusion coefficient (ADC). Colour-coded ROIs are correlated to the signal decay curves in (B) and (C). Diffusion intensity decay curves normalized to unity for representative regions of interest on large sized (B) and extra-large size (C) colony. (D) ADC map generated through Bruker internal ‘*dtraceb*’ algorithm. (E) Table of calculated ADC values for regions of interest shown in (A). Scale bar in A and D: 500 μm.

To resolve correlations between colony structure and diffusion dynamics in water and oil rich regions, apparent diffusion coefficients (ADC) in colonies were assessed with a series of images captured with incrementally increasing diffusion sensitising gradient strengths (Fig 4). Diffusion weighting works on the basis of twin dephasing gradient echoes with opposing direction. Because of this, only stationary protons are rephased completely. Protons that diffuse experience a net gradient and are dephased proportional to the distance travelled. In general, free water is most strongly dephased leading to a progressive darkening of background medium signal. Under a B-value of 1182 s mm^-2^, the water signal is already strongly diminished (Fig 4A). Remarkably, oil-rich regions in large colonies exhibited very low dephasing under strong gradients, as indicated by bright signal in these areas (S4 Fig and S1 Video). It implies that diffusion is highly restricted in oil rich regions, which appears to hold to a lesser extent for extra-large heterogeneous colonies as well.

Quantification of diffusion was achieved by calculation of apparent diffusion coefficients (ADC) for selected ROIs placed on two different types of colonies as shown in Fig 4A. A mono-exponential model for fitting shown in Fig 4B and 4C reflects the combined contributions of the water fraction and hydrocarbon fraction to the ADC. Because the hydrocarbon fraction is resistant to signal attenuation at the diffusion sensitising gradient strengths used (3123 s/mm^2^), it can be assumed to be constant for the purposes of mono-exponential curve fitting. Thus ADC reflects predominantly water diffusion. ADCs were also calculated at each voxel position for display in the ADC map depicted in Fig 4D. As summarized in tabular form in Fig 4E, the centre of a large size colony exhibited some restriction of diffusion 1.01 x 10^−3^ mm^2^ s^-1^ as compared to outside medium (2.4 x 10^−3^ mm^2^ s^-1^). The immediate area outside the central core (outer centre) was on average lower in diffusion at 0.87 x 10^−3^ mm^2^ s^-1^ corresponding to the thickest oil band observed in Fig 2C. The diffusion was found to be highest at the edge of colony (2.79x 10^−3^ mm^2^ s^-1^). This value was even higher than the values observed for the surrounding medium. This could possibly be due to enhanced relaxation rates related to the ‘halo’ phenomenon observed in *T*_*1*_ and *T*_*2*_ maps. Most anisotropy in diffusion was observed for the centre of extra-large colonies (Fig 4), while the region containing a fluid-filled interface was comparatively low in anisotropy.

Within the centres of extra-large heterogeneous colonies, a water channel exhibited an ADC value of 1.87x 10^−3^ mm^2^ s^-1^, interestingly higher than the large colony centre. Other parts of the interior exhibited low apparent diffusion coefficient (0.76x 10^−3^ mm^2^ s^-1^). Similar to the large sized colony, edge ADC of extra-large colonies is very high (2.69 x 10^−3^ mm^2^ s^-1^).

The ADCs are highly co-localised with the *T*_*1*_ relaxation characteristics shown in Fig 3B. The presence of interfaces between sub-colonies provides clues for possible anisotropy in diffusion, therefore six equally spaced diffusion directions were recorded, which also served to avoid background interference from imaging gradients in ADC calculation.

## Discussion

In this work, we provide the first non-invasive MRI means to analyse morphology and internal structural variation of *B*. *braunii* colonies and obtain spatial information of oil distribution, and diffusion dynamics *in vivo*. Large size colonies (700–1500 μm diameter) show a well-defined central core and number of surrounding layers. Previous studies indicate that the central core may be filled with the remainder of dead cells and their extracellular matrix [30]. If this is the case, it would stand to reason that oil would also be present here. However, this does not appear to be the case as illustrated by the absence of oil in colony centres measured by CSSI of oil resonances (Fig 1B). Though oil has been widely proposed as having important energy storage functions for algae, it has been reported that *B*. *braunii* is not capable of degrading its own long-chain hydrocarbons [31][32]. Thus, it is likely that algae do not store oil reserves in the centre, and oil found in the colonies is restricted either to the areas where living cells are localized and producing the oil or in the area immediate next to the living cell layer containing extracellular matrix. It is known that in large *B*. *braunii* colonies, living cells are exclusively located on outer rings of the colony [21]. This is in contrast to small *Showa* colonies (50–200 μm), where live cells are distributed in subcolonies throughout the entire colony [17]. Our CSSI, CSI as well as relaxations data clearly show that these outer ring areas of the colony contain high concentration of oil. The two concentric oil layers may be attributed to the complex extracellular matrix connecting individual cells [18,33]. This notion is in line with the low apparent diffusion coefficient observed on these oil containing rings (Fig 4).

In contrast to large colonies, the extra-large *B*. *braunii* colonies (1500–2500 μm diameter) show a significantly different internal makeup, namely an altogether more heterogeneous central part and less defined outer rings. These colonies correspondingly contain a more heterogeneous distribution of oil including at the central part of the colonies. The CSSI imaging of oil shows that less defined outer ring of these extra-large colonies is rich in oil content. The finding that oil is localised near the surface of both large colonies and extra-large colonies may have some implications for oil extraction from *B*. *braunii* colonies. For example, solvent-based extraction of oils for larger colonies could occur at similar efficiencies compared to smaller colonies, since the required penetration depth stays relatively equal (Fig 1B) [26].

One of the most important findings of this study is the observation of the existence of fluid channels, which could be visualized for the first time in live *B*. *braunii* colonies. In addition, by exploiting various MRI methods we were able to characterize the composition of these channels. However, the role of fluid channels in extra-large colonies is not clear. Since the network of water based fluid channels was found to be reaching to the surface of the colonies, its role in nutrient diffusion could be possible. The apparent diffusion coefficient measurements show that fluid channels have relatively low diffusion restriction as compared to other areas of the colonies.

The results of MSME imaging, relaxation and diffusion measurements also provide a clue toward nutrient and solvent exchange at the surface of colonies. The surface ring hyperintensity found in MSME imaging, is attributed to diffusion generated *T*_*1*_ contrast as it appears most strongly under short TR (≤1500ms) [34,35]. Hyperintense rings are known to arise from porous organic surfaces in contact with bulk water [34]. Although the thickness of the colony fibrillary sheath is known to be circa 4–7 μm, the resulting ring phenomenon is much larger, in the order of 50 μm (Fig 3) [18]. It can be postulated that this reflects a highly porous nature of the colony sheath to small molecules including water. Diffusion coefficient was found to be very high at the edge of the colony. This value was even higher than observed for surrounding medium. This could possibly be due to enhanced relaxation rates linked to the ‘halo’ phenomenon observed in *T*_*1*_ and *T*_*2*_ maps (Figs 3 and 4).

The extra-large heterogeneous colonies observed in this work may have been formed by the combination of several smaller colonies or by having multiple seed-colonies. Important to this argument is the knowledge that *B*. *braunii* is known to form biofilms as well as inclusion in bacterial biofilms [20,36]. This property is especially useful in Biofilm Photobioreactors (PBRs), which are seen as a promising avenue for commercialisation [37]. Linking of sub colonies together to form an extra-large colony is thus certainly plausible; possible in the life-cycle of *B*. *braunii*, and would provide a fast biomass accumulation scenario. Importantly, it provides a plausible mode of water channel formation, arising from interstitial space between growth clusters of *B*. *braunii*. These channels are particularly well visualised when viewed as a maximum intensity projection MIP, as seen in supplementary video (S2 Video). Curiously, segmentation of cells by retaining-wall shells is well known, but these shells are rich in hydrocarbons, not water [33]. Thus, our hypothesis may mean the organisational model for extra-large colonies is more complex than the current life cycle models of smaller colonies in literature. Our findings of colony organization are summarised graphically in S5 Fig. We conclude that imaging secondary metabolites directly and *in vivo* using MRI is feasible and provides an advantageous platform for the study of oleaginous algae and biofilms.

## Materials and methods

### *Botryococcus braunii* cultivation

*B*. *braunii*, variant Showa, was grown in a bubble column on a modified CHU-13 medium [38]. The medium was composed of CaCl_2_·2H_2_O (108 mg L^-1^), MgSO_4_·7H_2_O (200 mg L^-1^) and K_2_HPO_4_ (104.8 mg L^-1^), KNO_3_ (1.2 g L^-1^), Na_2_O_4_Se (9.44 mg L^-1^), FeNaEDTA (20 mg L^-1^). Micronutrients traces consisted of CuSO_4_ ·5H_2_O (0.08 mg L^-1^), ZnSO_4_·5H_2_O (0.19 mg L^-1^), CoSO_4_·7H_2_O (0.09 mg L^-1^), MnSO_4_·H_2_O (1.27 mg L^-1^), Na_2_MoO_4_·2H_2_O (0.06 mg L^-1^), H_3_BO_3_ (2.86 mg L^-1^) and concentrated H_2_SO_4_ (0.01 ml L^-1^). KNO_3_ concentration was chosen so as to minimise the potential of nitrogen growth limitations. Citric acid was omitted from the medium composition, ensuring phototrophic growth due to the absence of a carbon source. All ingredients were autoclaved separately and final medium was adjusted to pH 7.2 with NaOH. Cultures were transferred to fresh medium every 2 weeks to maintain exponential growth.

Culture illumination was provided by continuous cool LED lighting with a Correlated Colour Temperature (CCT) of 4300K and light intensity of 30 Klux or approximately 450 μmol s^-1^ m^-2^. Temperature was maintained at 25±1°C under continuous sparging with ambient air. To prevent evaporation due to gas bubbling, air was wetted by sparging through distilled water prior to entering the culture vessel. Cultures were grown in high light (450 μmol s^-1^ m^-2^) under continuous illumination for 15 weeks. Under these conditions colonies achieved remarkably large and extra-large sizes (700–2500 μm). For the MRI measurements, the colonies were transferred using a 2 ml volume pipet to a glass dish, drained of excess medium and then transferred by spatula to a 5mm NMR tubes. Teflon stoppers were inserted into the tubes to prevent displacement and dehydration of the colonies. Colonies were kept in culture medium during *in vivo* MRI measurements.

### MRI acquisition

All experiments were performed on a 17.6 T (750 MHz), vertical 89 mm wide bore magnet (Bruker Biospin, Ettlingen, Germany) in combination with an Avance I console. A Bruker Micro5 probe with 5mm birdcage resonator and a built-in 48 mT/m/A (1.92 T/m at 40 A) gradient system coupled to BAFPA 40A amplifiers was used for all measurements. All spectrometer operation was controlled by a Linux PC running Topspin 2.0PV and Paravision 5.1. Sample temperature was maintained at 293 ± 1 K through gradient water-cooling.

#### Multi Slice Multi Echo

Anatomical reference images were acquired using a Multiple Slice Multi Echo Sequence (MSME), refocusing magnetisation from an initial 90° pulse with a 180° pulse echo train. The following basic parameters were used for Fig 1A: echo train length = 4; All echo images were summed to produce a composite image. Average echo time (TE) = 13ms; Repetition time (TR) = 1500 ms; receiver bandwidth = 100 kHz; number of averages = 32; Field of View (FOV) = 5x5 mm^2^; matrix size = 256x256; and resolution = 19.5x19.5x100 μm^3^.

#### RAREVTR *T*_*1*_ and *T*_*2*_ mapping

Longitudinal (*T*_*1*_*)* and transverse (*T*_*2*_) relaxation maps were obtained using a Rapid Acquisition with Relaxation Enhancement with Variable TR (RAREVTR) pulse sequence. The sequence used a saturation scheme (i.e., varied TR) to acquire *T*_*1*_ and used a multi-echo CPMG scheme (i.e., varied TE) to acquire *T*_*2*_. The following parameters were used: TR-array = 200, 400, 800, 1500, 3000, 5500 ms; TE = 4.5, 9.0, 13.5, 18.0, 22.5, 27 ms for each TR; echo spacing = 4.5ms; RARE factor = 1; Number of averages (NA) = 16; matrix = 128x128; acquisition time = 4 hours 52 minutes. The field of view was 5x5 mm^2^ with a thin slice of 0.25 mm thick to prevent partial volume effects, resulting in a resolution of 39x39x250 μm^3^. The voxel volume was 3.8 · 10^−4^ mm^3^ and a receiver bandwidth of 100 kHz was used. A single slice was acquired to prevent interslice modulation effects. ROIs were manually defined using an Image Sequence Analysis tool package (ISA) (Paravision 5.1, Bruker), Transverse relaxation was calculated using the fit function: $M\left(t\right)=C^{\left(-\frac{t}{T_{2}}\right)}$, where C = signal intensity, and *T*_*2*_ = transverse relaxation time. The *T*_*1*_ values were determined by image sequence analysis using a fit function: $\text{M}\left(\text{t}\right)={\text{M}}_{0}\times \left(1-{\text{e}}^{\left(\frac{\text{t}}{T_{1}}\right)}\right)$, where M_0_ is the equilibrium magnetization. Built-in functions were used to generate *T*_*1*_ and *T*_*2*_ relaxation maps from the parameter fitting on a pixel by pixel basis.

#### Chemical Shift Selective Imaging

Chemical Shift Selective Imaging (CSSI) was used to acquire selective water and fat/oil images separated on the basis of biochemical composition, as opposed to differences in *T*_*2*_ in inversion-recovery methods [39]. A narrow bandwidth 90° Gaussian pulse was used for on resonance frequency selective excitation. FOV and Matrix were identical to MSME anatomical imaging: FOV = 5x5 mm^2^, Matrix = 256x256. Further basic parameters are as following: Receiver bandwidth = 100 kHz, TR = 1500 ms. Measurements were averaged 64 times for a total acquisition time of 6 hours 49 minutes. Two separate measurements were performed with 1500 Hz and 600 Hz of excitation bandwidth respectively. The water oil frequency difference was determined to be 2.7 kHz at 17.6T. Signal cross contamination was prevented by choosing a bandwidth of 1.5 kHz, covering the whole oil containing spectral region.

For Fig 1B and 1C, Excitation bandwidth = 1500 Hz, -3.00 ppm. Slice thickness = 500 μm; resolution 19.5x19.5x500 μm^3^ and TE = 6.2 ms. Number of averages of Fig 4B = 220. For Fig 2B and 2C, Excitation bandwidth = 600 Hz, oil excitation was offset to 1.75 ppm. Slice thickness = 250 μm; resolution = 19.5x19.5x250 μm^3^ and TE = 8.3 ms.

#### Chemical Shift Imaging

Chemical Shift Imaging (CSI) was utilised in spin echo slab selective mode. CSI employs two orthogonal phase encoding steps with pulsed gradients to record a pure spectroscopic echo upon acquisition, instead of a conventional readout gradient used in imaging. A Hanning function weighted *k*-space acquisition scheme was utilised, as implemented by the Bruker *‘weighted’* measuring method, for an improved Spatial Response Function (SRF). Basic parameters were as follows: TR = 1100 ms; TE = 12 ms; Matrix = 32x32; FOV = 5x5 mm^2^; slice thickness = 250 μm; resolution = 156x156x250 μm^3^; number of scans = 45,000. Data was reconstructed into a 64x64 matrix with linear smoothing for display. Excitation and refocusing was achieved using Sinc3 pulses with a bandwidth of 8000 Hz. Echoes were captured into 2048 points in 204.80 ms, spectral resolution = 2.4 Hz per point; and Spectral width = 10 kHz (13.3 ppm). Magnetic field homogeneity in the selected volume was optimized by shimming the water resonance. A VAPOR suppression scheme of 625 ms was applied for efficient water signal saturation. Interpulse radiofrequency delay was 150, 80, 160, 80, 100, 37.2, 15 ms between seven hermite shaped CSSI modules. RF bandwidth = 900 Hz, excitation offset = -75 Hz (-0.1 ppm).

### Diffusion weighted MRI

Diffusion measurements were carried out with a spin-echo pulse sequence containing a pair of mono-polar diffusion-sensitising gradients. Gradient orientations were isotopically distributed in six directions. Gradient strength ranged from 14, 406, 1182, 1723, 3123 s/mm^2^ effective B-values, including imaging gradients. Diffusion gradient duration (δ) of 2.5 ms was combined with 5 ms diffusion gradient separation (Δ), for a total TR and TE of 1500 and 10.1 ms respectively. To obtain sufficient SNR 16 averages were recorded resulting in a total acquisition time of 16 hours. FOV was 5x5 mm^2^, matrix size 96x96 and slice thickness of 0,5 mm, resulting in a resolution of 52x52x500 μm^3^. Receiver BW was 100 kHz.

### Post-processing and analysis

All experimental data were acquired and processed using Paravision 5.1 (Bruker Biospin, Ettlingen, Germany) running on CentOS 3 and figures were prepared in Adobe Photoshop CC 2015.3 and Adobe Illustrator CC 2015.2 (Adobe Systems Incorporated, Mountain View, California, USA). A false colour image (S3 Fig) was generated by overlaying CSSI Oil signal on CSSI Water signal using the *‘lighten’* transfer mode.

#### 3D Volume reconstruction models

3D-MSME data were exported to DICOM using Paravision 5.1, consequently reconstructed in Slicer 3D 4.8 (www.slicer.org, [40]) and further processed in Meshmixer (Autodesk, San Rafael, California, USA).

#### Processing of CSI data

Integration of selected signal areas in magnitude mode were overlaid on MSME reference spectra using the Bruker CSI Visualisation Tool. A representative 1D spectrum was processed and exported using CSI-Tool (Bruker).

#### Processing of diffusion data

Diffusion data was processed with a trapezoid windowing function to remove DC offset artefact (window maximum between 12.5% and 87.5% of acquisition window). Windowed results were consequently analysed using the Bruker Image Sequence Analysis Tool. Signal intensity and standard deviation were derived from the internal fitting function ‘dtraceb’: $S\left(b\right)=A+Ie^{Db}$ Where *A is* an Offset and *I* the amplitude of diffusion with diffusion coefficient *D*. Several Regions of Interest (ROI) were selected and used for further calculations. Tabulated data containing ROI decay curves was exported for further analysis. Because the system contains two fraction, i.e., water (*w*) and hydrocarbons (*h*), normally bi-exponential fitting is required to accurately describe the system: $S\left(b\right)=f_{w}e^{-bD_{w}}+f_{h}e^{-bD_{h}}$ Where $f_{h}=1-f_{w}$. However, because the maximum gradient strength used was only 3123 s/mm^2^, oil is not significantly attenuated because: $b\ll \frac{1}{D_{h}}$. This means that the diffusion attenuation for the oil/hydrocarbon fraction simplifies to: $e^{-bD_{h}}\approx 1$. As a consequence the system can then be described mono-exponentially as follows: $S\left(b\right)=f_{w}e^{-bD_{w}}+f_{h}$. The mono exponential fitting was performed in OriginPro 9.1.0 with Levenberg-Marquardt algorithm iteration (OriginLab Corporation, Northampton, Massachusetts, USA).

## Supporting information

S1 Fig Pseudo-3D MSME successive axial slices through the colonies showing variation in size and structure of *B*. *braunii* colonies. Imaging parameter used (TR, 1500 ms; TE, 13 ms; acquisition time, 3h24m; Average, 32). Resolution 19.5x19.5x200 μm^3^ captured with a matrix of 256x256. Scale bar: 1000 μm
(TIF)

S2 Fig CSSI of water resonances. Imaging parameters used are: repetition time, 1500 ms; echo time, 8.32 ms; number of averages, 64 and resolution, 19.5x19.5x250 μm3. Receiver bandwidth used was 100 kHz. Excitation pulse of 600 Hz wide at water resonance. Inhomogeneity in the distribution of water in the centre is seen (arrowhead). The water signal was found to be low in two oil containing bands (arrows). Scale bar: 500 μm.
(TIF)

S3 Fig False colour overlay of oil/lipid signal in red and water signal in blue. An enlarged area of extra-large colony in (A) is shown in (B) depicting a possible septum (white arrow). Scale bar 500 μm (A), 100 μm (B).
(TIF)

S4 Fig Diffusion weighted images captured for ADC Mapping. Individual images from the +Z diffusion weighting direction. TR, 1500 ms; TE, 10.1ms; 16 averages; diffusion gradient duration 2.5 ms and gradient separation of 5 ms; effective B-values range: 14, 406, 1182, 1723, 3123 s/mm2. Scale bar 500 μm.
(TIF)

S5 Fig Summarizing model of *B*. *braunii* colony organisation of large colony (700μm-1500 μm) (left) and extra-large colony (>1500 μm) (right). Boundary model derived from integration of MSME and CSSI imaging results on morphology of the colonies seen in Fig 2.
(TIF)

S1 Video Animated movie of diffusion weighting steps. Movie version generated form S4 Fig. Video length 6s, 251 frames. Scale bar 500 μm.
(MP4)

S2 Video Maximum intensity projection of 3D MSME imaging. Maximum Intensity Projection Video (MIP) of 3D-MSME data of multiple colonies submerged in perfluordecalin (PFD), which highlights the difference in core structure of large and extra-large colonies. Processed in Slicer 3D. Time between repetitions 350 ms and echo-time 4.66 ms. Time of acquisition 4d12h18m, 29 averages. Resolution 23x23x23 μm^3^ captured with a matrix of 256x196x196. Video length 10s, 360 frames.
(MP4)
