# Supplementary figures and images for: Non-invasive magnetic resonance imaging of oils in Botryococcus braunii green algae: Chemical shift selective and diffusion-weighted imaging
Source: PLoS One. 2018 Aug 30;13(8):e0203217. doi: 10.1371/journal.pone.0203217 (PMC6117053; doi:10.1371/journal.pone.0203217)

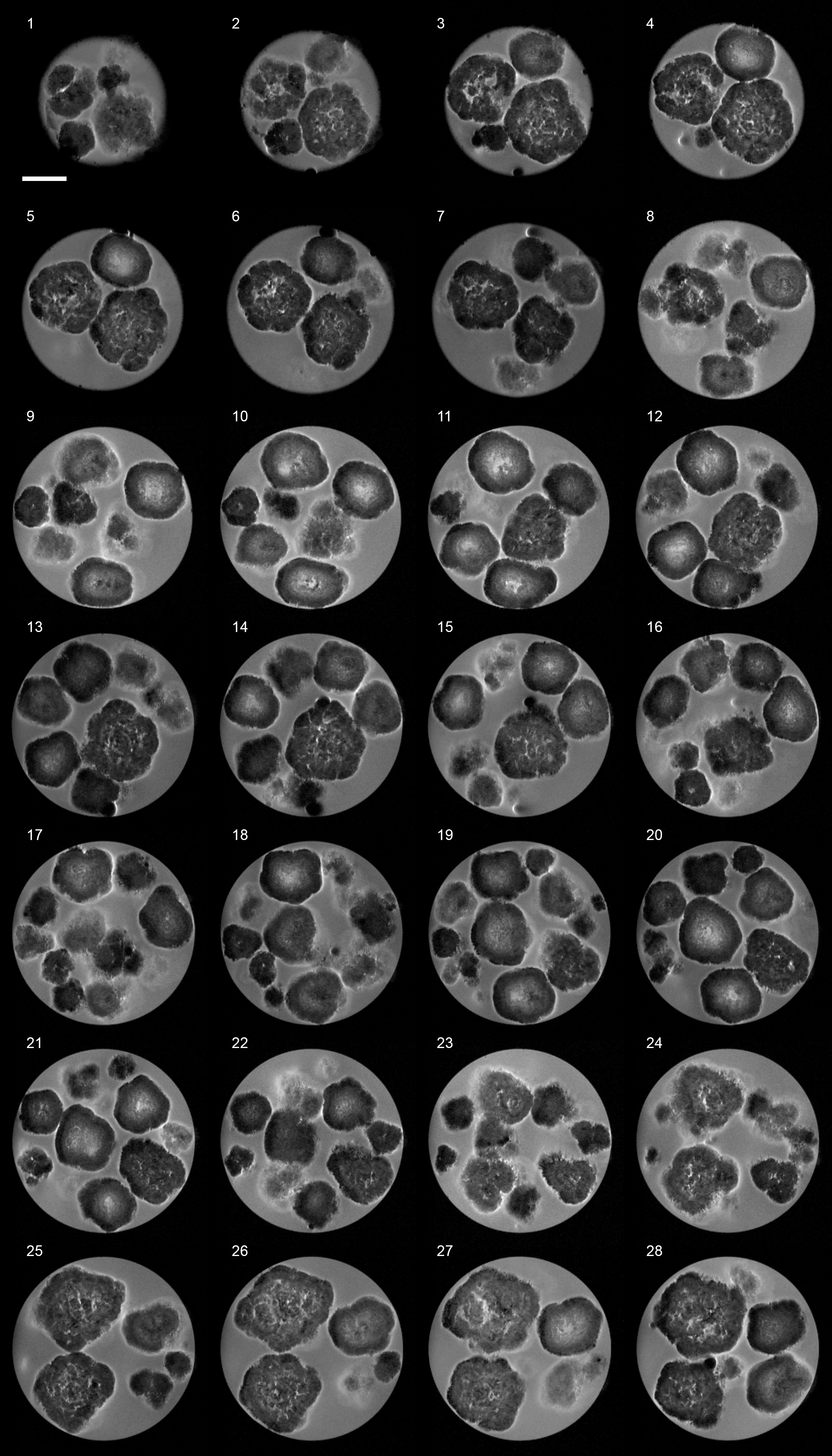

Supplement: S1 Fig — Imaging parameter used (TR, 1500 ms; TE, 13 ms; acquisition time, 3h24m; Average, 32). Resolution 19.5x19.5x200 μm3 captured with a matrix of 256x256. Scale bar: 1000 μm (TIF) [file pone.0203217.s001.tif]

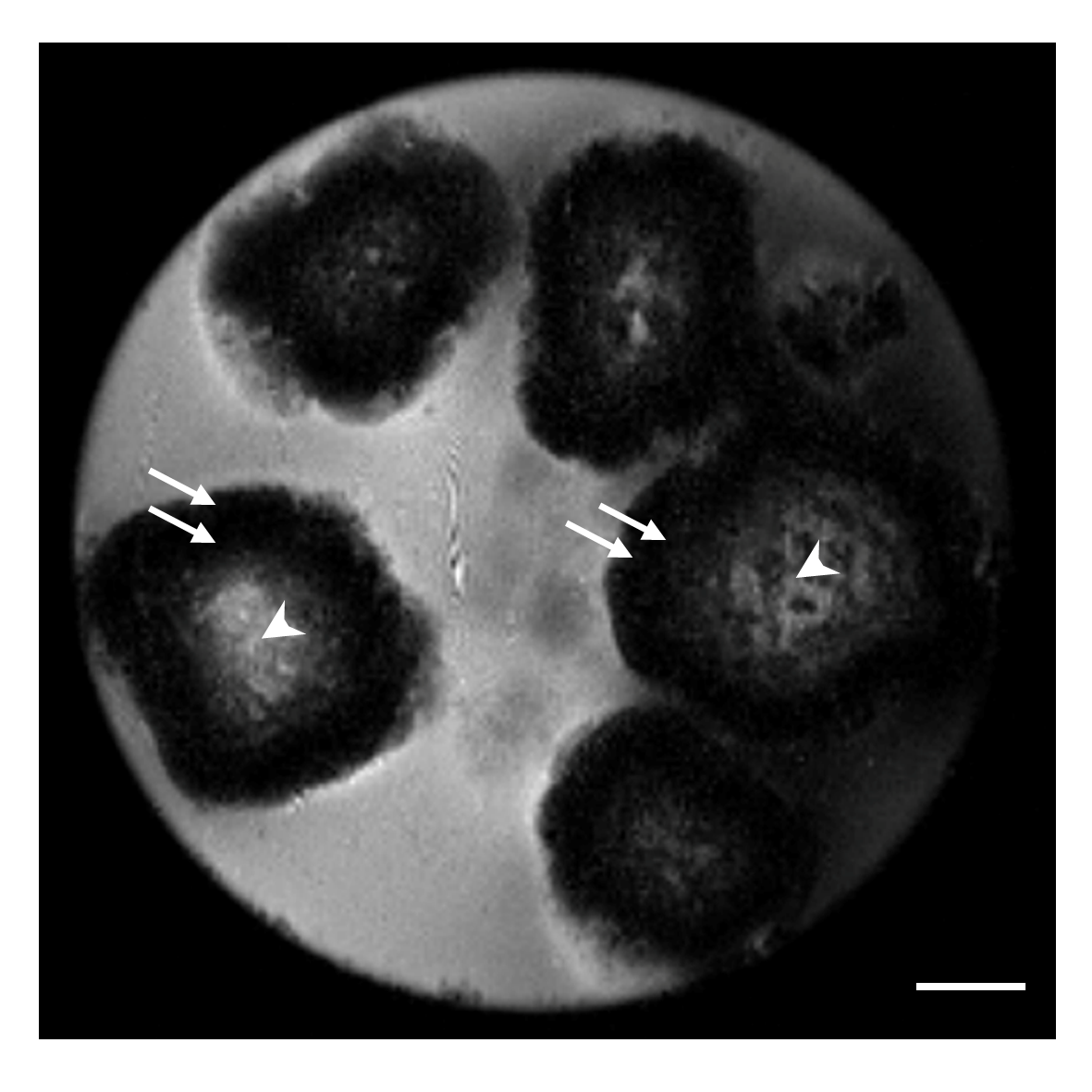

Supplement: S2 Fig — Imaging parameters used are: repetition time, 1500 ms; echo time, 8.32 ms; number of averages, 64 and resolution, 19.5x19.5x250 μm3. Receiver bandwidth used was 100 kHz. Excitation pulse of 600 Hz wide at water resonance. Inhomogeneity in the distribution of water in the centre is seen (arrowhead). The water signal was found to be low in two oil containing bands (arrows). Scale bar: 500 μm. (TIF) [file pone.0203217.s002.tif]

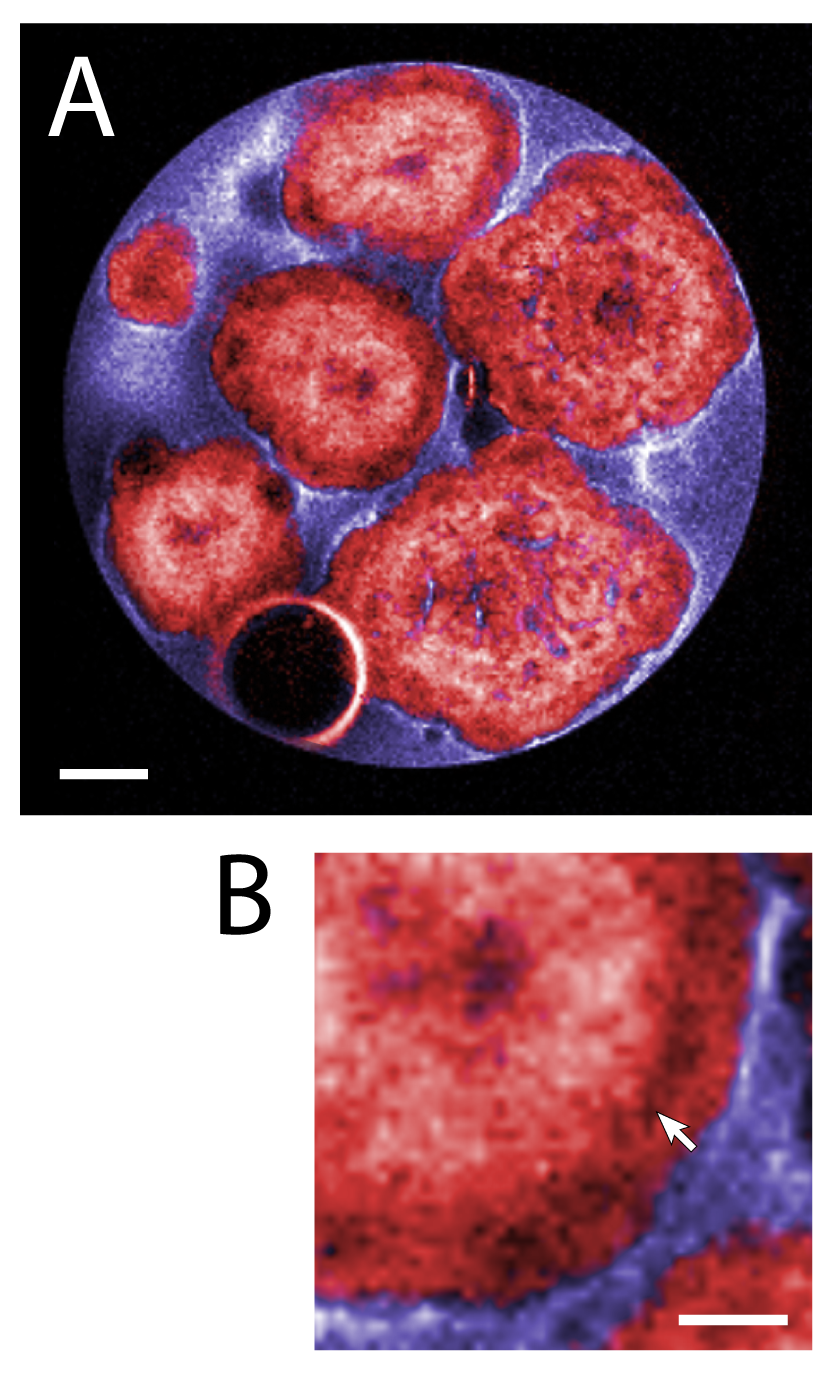

Supplement: S3 Fig — An enlarged area of extra-large colony in (A) is shown in (B) depicting a possible septum (white arrow). Scale bar 500 μm (A), 100 μm (B). (TIF) [file pone.0203217.s003.tif]

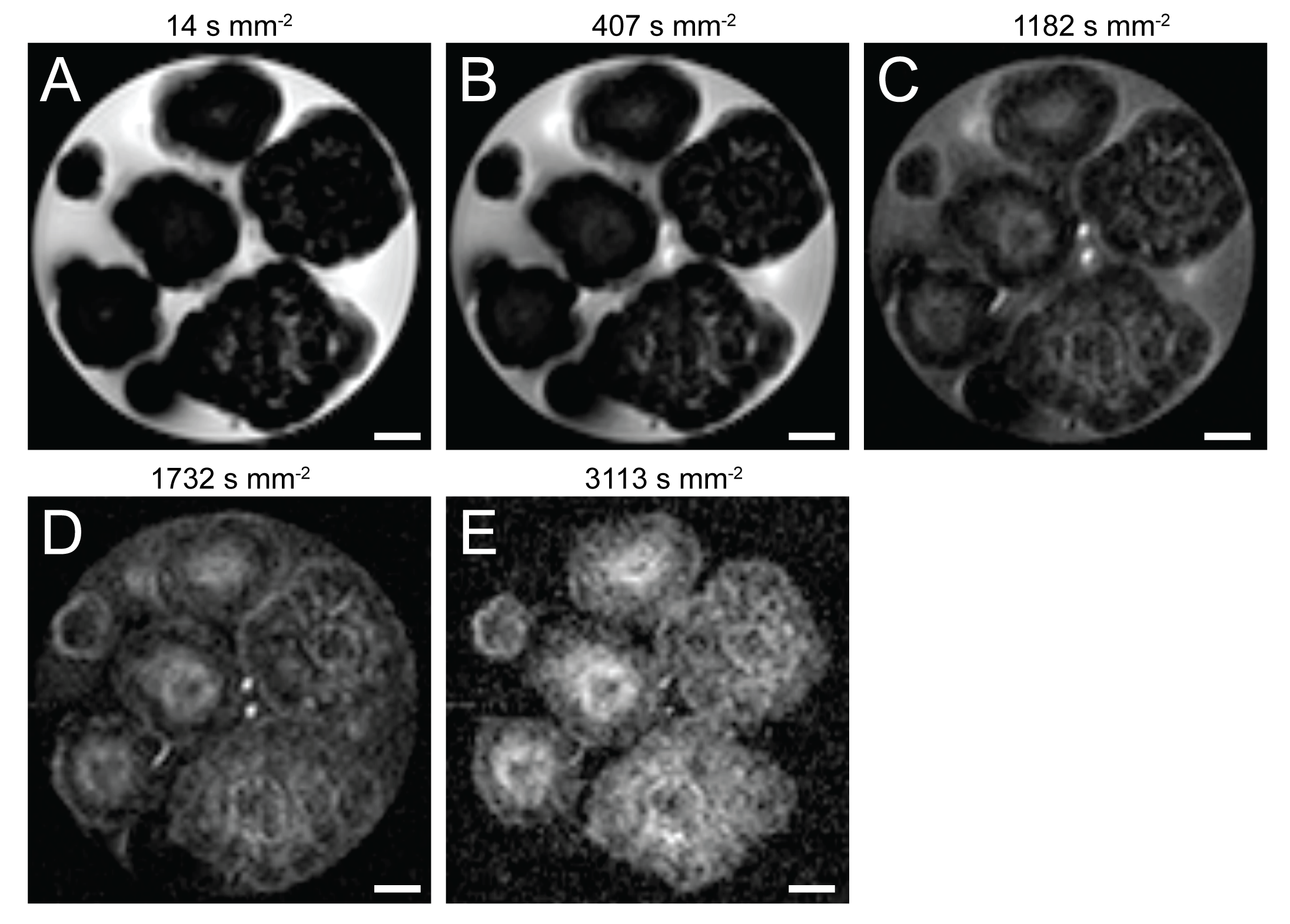

Supplement: S4 Fig — Individual images from the +Z diffusion weighting direction. TR, 1500 ms; TE, 10.1ms; 16 averages; diffusion gradient duration 2.5 ms and gradient separation of 5 ms; effective B-values range: 14, 406, 1182, 1723, 3123 s/mm2. Scale bar 500 μm. (TIF) [file pone.0203217.s004.tif]

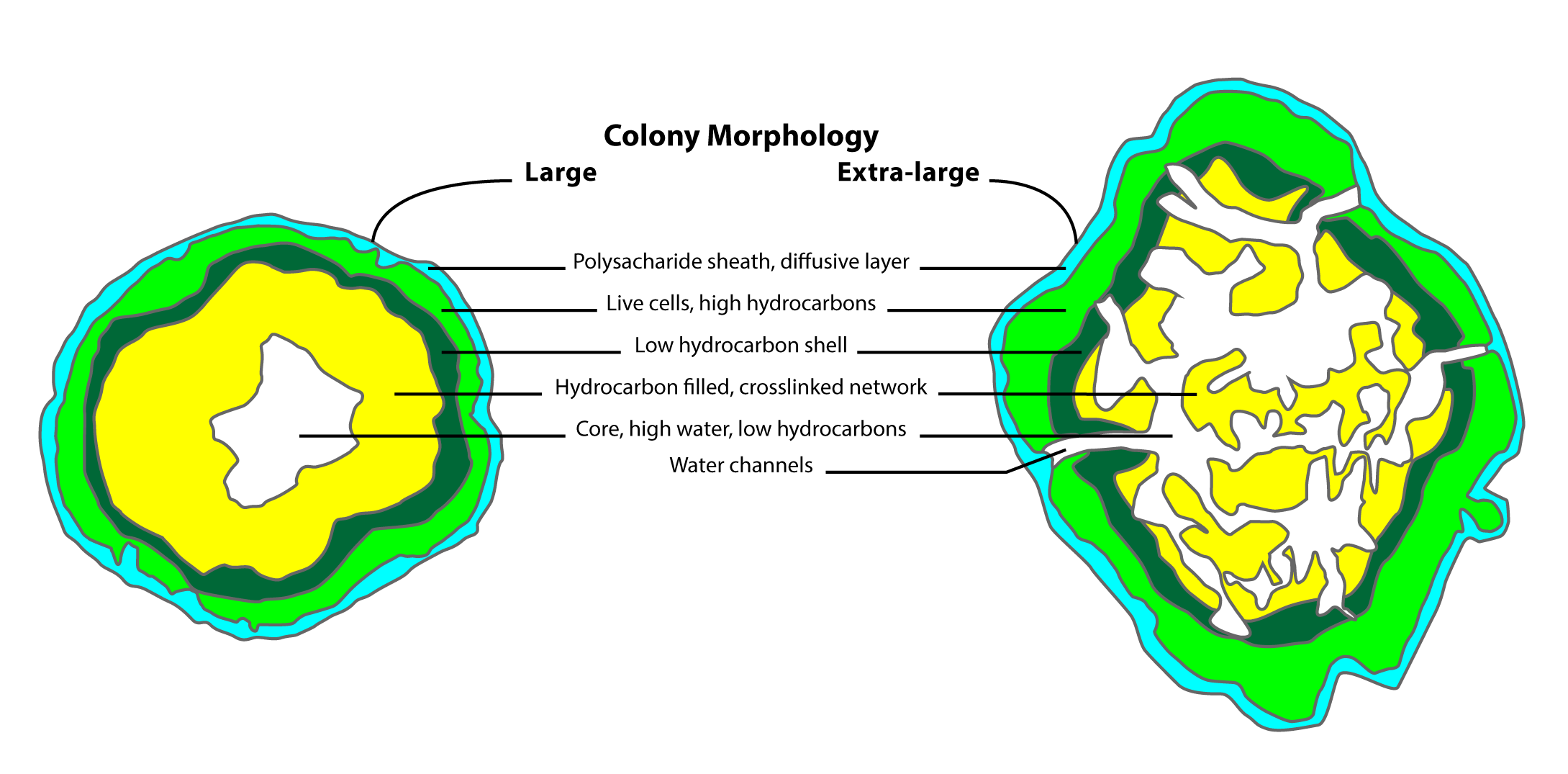

Supplement: S5 Fig — Boundary model derived from integration of MSME and CSSI imaging results on morphology of the colonies seen in Fig 2. (TIF) [file pone.0203217.s005.tif]
